# Fluorescence lifetime imaging for studying DNA compaction and gene activities

**DOI:** 10.1038/s41377-021-00664-w

**Published:** 2021-11-02

**Authors:** Svitlana M. Levchenko, Artem Pliss, Xiao Peng, Paras N. Prasad, Junle Qu

**Affiliations:** 1 Key Laboratory of Optoelectronic Devices and Systems of Ministry of Education and Guangdong Province, College of Physics and Optoelectronic Engineering, Shenzhen University, Shenzhen, Guangdong 518060 China; 2 Department of Cell Biophysics, Faculty of Biochemistry, Biophysics and Biotechnology, Jagiellonian University, 30-387 Krakow, Poland; 3 Institute for Lasers, Photonics and Biophotonics, University at Buffalo, State University of New York, Buffalo, NY 14260-3000 USA

**Keywords:** Biophotonics, Imaging and sensing

## Abstract

Optical imaging is a most useful and widespread technique for the investigation of the structure and function of the cellular genomes. However, an analysis of immensely convoluted and irregularly compacted DNA polymer is highly challenging even by modern super-resolution microscopy approaches. Here we propose fluorescence lifetime imaging (FLIM) for the advancement of studies of genomic structure including DNA compaction, replication as well as monitoring of gene expression. The proposed FLIM assay employs two independent mechanisms for DNA compaction sensing. One mechanism relies on the inverse quadratic relation between the fluorescence lifetimes of fluorescence probes incorporated into DNA and their local refractive index, variable due to DNA compaction density. Another mechanism is based on the Förster resonance energy transfer (FRET) process between the donor and the acceptor fluorophores, both incorporated into DNA. Both these proposed mechanisms were validated in cultured cells. The obtained data unravel a significant difference in compaction of the gene-rich and gene-poor pools of genomic DNA. We show that the gene-rich DNA is loosely compacted compared to the dense DNA domains devoid of active genes.

## Introduction

Studies of the cellular molecular structure have traditionally relied on fluorescence microscopy approaches including conventional laser scanning confocal microscopy^1^ and multiphoton imaging^2,3^. With the advent of powerful STORM (stochastic optical reconstruction microscopy), PALM (photo-activated localization microscopy), and STED (stimulated emission depletion microscopy) super-resolution imaging techniques^4–6^, the cellular architecture was visualized in unprecedented details, bringing about a revolution in cell biology research. However, it is still challenging to study intracellular distribution and interactions of biomolecules due to the limited spatial resolution of fluorescence microscopy. While the lateral resolution of the super-resolution techniques is approaching 10 nm, the axial resolution still lags behind^7,8^, which is a bottleneck in the studies of 3D geometry of complex intracellular structures.

An alternative strategy that overcomes existing limitations of conventional approaches for exploring subcellular structure at nanoscale involves fluorescence lifetime imaging (FLIM)^9–12^. Briefly, FLIM is a technique that maps the spatial distribution of the fluorophore lifetimes within microscopic images. A notable advantage of FLIM is that fluorescence lifetime values are not affected by changes in probe concentration, excitation intensity, and photobleaching, thus enabling more robust and reproducible measurements than fluorescence intensity imaging^13^. The most common application of FLIM in biology exploits the Förster resonance energy transfer (FRET) mechanism, designed for probing close molecular interactions^13^. In FLIM-FRET, the nonradiative energy transfer from a donor to an acceptor fluorophore can occur only when both fluorophores are in close proximity to each other (<10 nm), causing depopulation of donor excited electronic states. As a result, the donor fluorescence lifetime is decreased, while the acceptor’s lifetime is increased, which enables the detection of spatial association between the fluorophores^14^. In addition to FRET, the fluorescence lifetime of a fluorophore is responsive to the refractive index (RI) of its microenvironment. This dependence is an inverse quadratic correlation between the lifetime of the fluorophore and the local RI of its environment, determined by the Strickler-Berg equation proposed several decades ago^15^. More recently, the sensitivity of fluorescence lifetimes of fluorophores to intracellular local RI was experimentally verified^16–19^; for review see^20^.

Here, we report on the development and application of FLIM approach to collect new layers of information for studies of 3D cellular architecture, with a specific example of genomic structural organization, which belongs to the most exciting and challenging problems in fundamental cell biology. The complexity of this field stems from a high degree of compaction required to accommodate ~2 m of genomic DNA into the cell nucleus, which typically measures only five to ten micrometers in diameter. Moreover, the packing of DNA is highly irregular, wherein non-coding DNA segments are often found in densely packed domains, while gene-rich sequences maintain relatively relaxed configuration to provide access to soluble proteins catalyzing RNA synthesis and post-transcriptional modifications^21,22^.

Studies of the last decades have elucidated a number of basic principles in DNA compaction. In general, DNA polymer associates with a set of DNA-binding proteins into a mass of convoluted fibers collectively known as chromatin. Classic studies identify several hierarchical levels in DNA compaction starting from wrapping of the DNA strand around nucleosomes, made of core histone proteins, and giving rise to “beads on a string” fiber, ~10 nm thick^23^. These first-order DNA-protein fibers are believed to condense into gradually thicker filaments, albeit these structures remain poorly understood^24–27^. At the next hierarchical level, thick chromatin filaments fold into large loops, which in turn form chromatin domains (CD) measuring slightly over 100 nm and containing close to a million of DNA base pairs^28,29^. The CDs are viewed as structural units for the regulation of DNA compaction, maintenance, replication, and gene expression. At the same time, the compaction density of the chromatin in CD is not static, but dynamically fluctuates over time^30^. Apparently, these dynamic changes are linked to genes activity. It was found that the pool of CDs, which replicate at the beginning of the S-phase of the cell cycle and are known to contain active genes are far more dynamic than CDs which replicate later in the S-phase and contain little if any active genes^30,31^. Moreover, continuous changes in chromatin compaction contribute to genomic functions, including regulation and coordination of genes expression, DNA replication, and repair^22,32,33^. Notably the variations in compaction are unique for individual genomic loci^34,35^, which highlights the importance of high-resolution mapping of chromatin structure.

Nevertheless, studies of the geometry of chromatin fibers, compaction, and dynamic reorganization during physiologic processes or disease development in live-cell environment have been exceedingly challenging. Currently, most of our knowledge on DNA packing has been obtained by in vitro experiments on isolated DNA polymer and DNA-binding proteins or by theoretical simulations^36^. These studies unraveled physico-chemical mechanisms underlying the formation of chromatin fibers and generated quantitative models of DNA compaction. However, the data obtained in test-tubes are hard to verify in native cellular structures, thus the models of chromatin organization are mostly hypothetical^27,32,37,38^.

Remarkably, applications of FLIM open new directions in the analysis of chromatin compaction at the nanoscale in either live or fixed cells. Up-to-date, several studies adopted the FRET mechanism, in which energy transfer occurs between the donor and acceptor fluorophores linked to the DNA polymer. In this system, the chromatin condensation causes an increase of FRET, and in turn shortening of the donor’s lifetime, while the chromatin relaxation reduces the probability of FRET, resulting in a corresponding lifetime increase^39^. This assay was adapted for studies of chromatin density within chromosomes of live cells throughout mitosis^12^. More recently, FLIM was applied for probing the chromatin condensation state using membrane-permeable, DNA-binding fluorophores (e.g., Hoechst 33342 and PicoGreen) or histone tagged fluorescent proteins (e.g., EGFP-H4)^39,40^.

However, the DNA dyes and fluorescence-tagged histones associate with the entire chromatin in the cell nucleus. Hence, this traditional approach provides no direct information on the compaction of any specific pool of DNA, such as gene-rich or non-coding sequences. Furthermore, when applied to DNA-binding proteins such as histones, the FLIM-FRET technique does not reach its full potential by averaging the data at the level of nucleosomes and not directly interrogating the structure organization of DNA strands. Another limitation is that nucleosomes are not typically present in actively transcribed chromatin and thus FRET signal could only be used to study compaction of non-coding sequences and inactive genes. To address this issue, we propose the development of two independent assays, wherein fluorescence-labeled nucleotides are directly incorporated into the DNA strands during the S-phase of the cell cycle and their lifetimes convey changes in DNA compaction.

The first proposed FLIM-based assay utilizes the quadratic dependence between fluorescence lifetime and local RI, - responsive to the DNA compaction density discussed above. Another FLIM approach employs FRET between the fluorescence-labeled nucleotides incorporated into the DNA strands. Compared to FLIM of fluorescence-tagged nucleosomes^12^, the advantage of DNA labeling techniques is twofold. First, they enable selective labeling of either gene-rich or non-coding DNA sequences, which replicate at different windows of the S-phase^28^. Hence compaction of specific fractions of chromatin could be comparatively analyzed. Second, these techniques directly interrogate the DNA polymer compaction, independent of proximity between nucleosomes, and therefore may yield a higher sensitivity compared to previously developed approaches. Finally, the FLIM imaging of replication-labeled DNA could be combined with super-resolution microscopy. This combination was already validated for super-resolution modalities utilizing pulsed scanning lasers and single-point detection, such as STED^41,42^, while the integration of FLIM with widefield, single-molecule localization modalities, such as STORM, is currently underway. Such multimodal approaches may potentially enable monitoring of DNA compaction at the nanoscale during basic genomic processes including DNA replication and RNA synthesis.

## Results

### FLIM approaches to measurements of chromatin compaction

#### (I) RI as a function of DNA compaction

The electron and light microscopy observations have long identified that the distribution of proteins in genomic chromatin is non-uniform and correlates with the DNA compaction levels, producing densely packed heterochromatin domains with high RI^43^. It is worth noting that the DNA and proteins have a comparable contribution to RI of chromatin, since both types of biomolecules are present in a close mass ratio ranging, by different estimates, between 1:1 and 1:2 for DNA and proteins respectively^44^. In addition, proteins and DNA have close refractive increments, which is the dependence of RI on solute concentration^45^. Since variations in the concentration of proteins and DNA invariably influence the local RI, it is tempting to probe chromatin compaction levels by using the FLIM approach. As discussed above, the Strickler-Berg equation predicts the shortening of fluorophore lifetime with an increase of local RI. To validate the sensitivity of FLIM for this task we exploited a well-established correlation between the density of DNA packing and its replication timing, ranging from relaxed euchromatin replicating in the early S-phase to densely packed heterochromatin clumps, which replicate in the late S-phase. In these experiments, the cells were synchronized with aphidicolin, an inhibitor of DNA polymerase, at the border between G1- and S-phases^46^. Then, following the release from aphidicolin block, cells synchronously entered the S-phase and were incubated with bromodeoxyuridne (BrdU)—a halogenated nucleotide, which readily incorporates into the newly synthesized DNA strands. The halogenated nucleotides permanently stay in genomic DNA, and are accessible to fluorescence probes as the cell progresses through the cell cycle and into subsequent generations. The moderate amount of BrdU, which incorporates during the several minutes pulse, does not visibly alter the genomic functions or cell cycle progression^28^. Furthermore, BrdU does not perturb chromatin ultrastructure even at the electron microscopy level^47^. The protocol was adjusted to target either early, mid or late S-phase replicating chromatin, followed by immunofluorescence labeling of the BrdU incorporated into the DNA, as described in “Methods”.

**Fig. 1:**
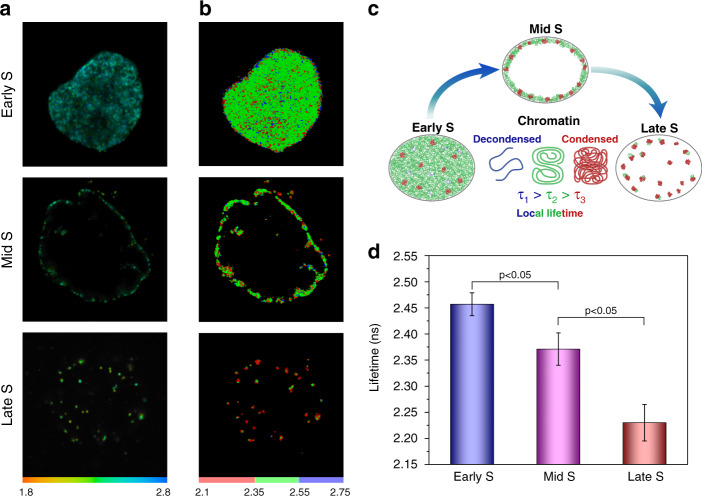
Dependence of fluorophore’s lifetime on chromatin replication timing. **a** Representative fluorescence lifetime images of early, mid, and late S-phase DNA replication sites labeled with AlexaFluor 546. Corresponding lifetime histograms are demonstrated in Fig. S3. **b** Color-coded image of the lifetime distribution throughout cell nucleus. **c** Schematics of the FLIM approach to measure compaction of chromatin replicating at different S-phase windows. **d** Averaged fluorescence lifetime of AlexaFluor 546 used to label DNA replication sites in early, mid, and late S-phase. The error bars correspond to standard deviations. See Fig. S4 for additional examples

Consistent with the synchronization procedure, labeled DNA replication sites were arranged into canonical early, mid or late S-phase patterns^48,49^. In the early S-phase, numerous replication sites were scattered throughout the nucleoplasm, while in the mid and the late S-phase, labeled sites were fewer, clustered together, and frequently located at the nucleolar and nuclear periphery (Fig. 1a). Remarkably, we documented a significant shortening of the fluorescence lifetime throughout the transition from the early to mid and late S-phase (Fig. 1d), while the fluorescence intensity of the replication signal did not visibly change. The averaged lifetime values were 2.46, 2.37, and 2.23 ns in early, mid, and late S-phase windows, correspondingly.

To illustrate changes in chromatin compaction at different stages of S-phase, measured lifetimes were color-coded into three ranges: short (2.1–2.35 ns, red), intermediate (2.35–2.55 ns, green), and long (2.55–2.75 ns, blue). The segmentation of lifetime was performed empirically based on the lifetime distributions of AlexaFluor 546 labeled genomic DNA in different stages of S-phase that allow optimal data visualization. Using this color assignment, the late S-replicated replication sites are almost exclusively colored red, while early and mid S-phase replication sites were predominantly green, with the inclusion of red and blue colored domains (Fig. 1b). Overall, the FLIM approach was sensitive enough to detect and measure differences in compaction between relaxed euchromatin fibers replicating in early S-phase and compacted heterochromatin domains replicating in mid to late S-phase.

**Fig. 2:**
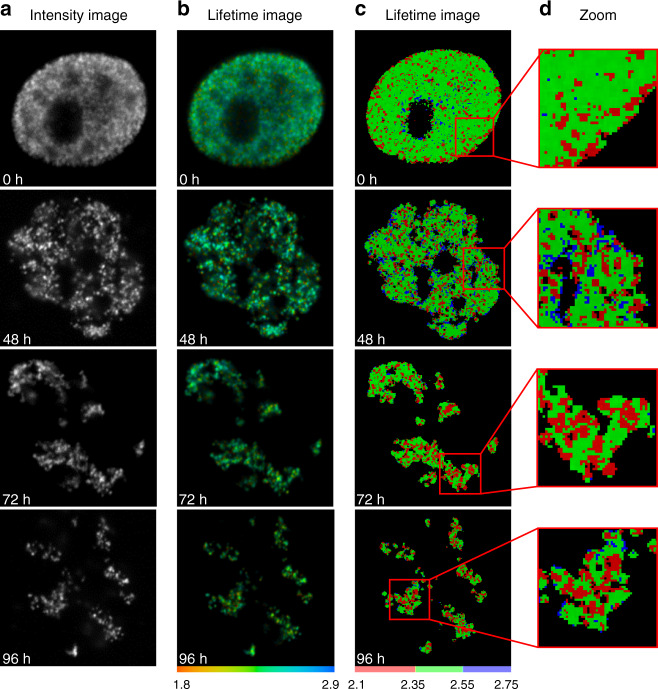
FLIM imaging of labeled chromosome territories. Synchronized in early S-phase cells were pulse-labeled with BrdU and chased through subsequent cell cycles to yield segregation of labeled chromosome territories. Representative fluorescence intensity (**a**), continuous (**b**), and discrete (**c**, **d**) fluorescence lifetime images are shown. See Fig. S5 for additional examples

In the next series of experiments, we studied the chromatin compaction beyond the S-phase of the cell cycle. Upon completion of replication, the pattern of labeled CDs is maintained as the cells progress through the cell cycle and into subsequent generations^28^. In these cells, we further evaluated the sensitivity of FLIM approach to variations in chromatin compaction density. Here the cells synchronized in early S-phase were exposed to BrdU for five minutes, and placed in a fresh medium for long-term incubation. Several cell divisions after the treatment, the BrdU label was confined to a subset of chromosomes segregated with unlabeled areas, as a result of new DNA strands synthesis in accordance with semiconservative mechanism of DNA replication^30^. The label segregation enables visualization of individual chromosome territories, which begin to emerge at 48 h following the exposure to BrdU. In the cells grown for 96 h after the BrdU pulse, the labeled chromosome territories appear to be completely separated from the rest of the nuclear volume, consistent with previous reports^30,50^ (Fig. 2). The segregation of labeled chromosome territories reduces the signal crowding, and facilitates the image analysis.

Similar to experiments with active replication sites, we found that the fluorescence lifetime of the AlexaFluor 546 was not uniform, ranging from ~2.1 to 2.75 ns. The bulk of the fluorofore linked to early S-phase replicated chromatin was in the 2.35–2.55 ns range, indicating an intermediate density of chromatin compaction (Fig. 2c, green color). A significant fraction of chromatin was associated with short lifetime of fluorofore (red color), these areas indicative of higher RI were often surrounded by the green-colored chromatin with the intermediate lifetime. Finally, minor pools of chromatin, typically localized at the periphery of segregated chromosome territories were showing relatively long lifetime (2.55–2.75 ns), expected for the relaxed chromatin structure, as indicated by blue color on the FLIM maps (Fig. 2c, d). Interestingly, in previous studies, it has been indicated that gene-rich DNA sequences tend to localize at the periphery of the chromosome territories^51^. Our data support earlier reports and further indicate that chromosome boundaries are enriched in loosely compacted chromatin fibers.

#### (II) FRET mechanism for probing DNA compaction

**Fig. 3:**
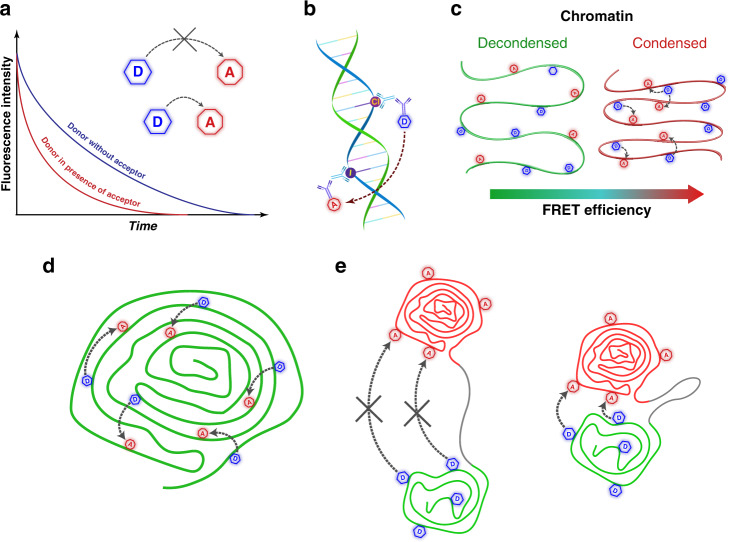
Schematic illustration of the FLIM-FRET assay to study genomic DNA compaction. **a** Basic FLIM-FRET mechanism. **b** Direct labeling of the genomic DNA by incorporation of fluorophore-tethered nucleotides. **c** Relation between chromatin condensation levels and FRET efficiency. Scheme of simultaneous (**d**) and sequential labeling of the chromatin domains (**e**) during the S-phase of the cell cycle

To explore the suitability of FLIM-based approach for probing DNA compaction at the nanoscale level, we employed a more comprehensive FLIM-FRET technique. As proposed here, the single-strand DNA labeling approach involves double labeling of newly synthesized DNA with halogenated nucleotides, such as Chloro-deoxyuridine (CldU) and Iodo-deoxyuridine (IdU), which incorporate into the same strand of DNA and could be selectively detected by immunofluorescence staining^52^. When CldU and IdU are labeled by a donor-acceptor pair of fluorophores the energy transfer from donor (D) to acceptor (A) molecules leads to the shortening of donor’s lifetime value^13^ (Fig. 3a, b). While this technique involves sophisticated double labeling protocols, it offers inherently higher sensitivity to variations in DNA compaction than RI mapping by FLIM (Fig. 3c). Moreover, incorporation of D and A fluorophores into DNA segments replicating at different S-phase windows, could elucidate co-compaction of sequentially replicated DNA segments (Fig. 3d, e).

**Fig. 4:**
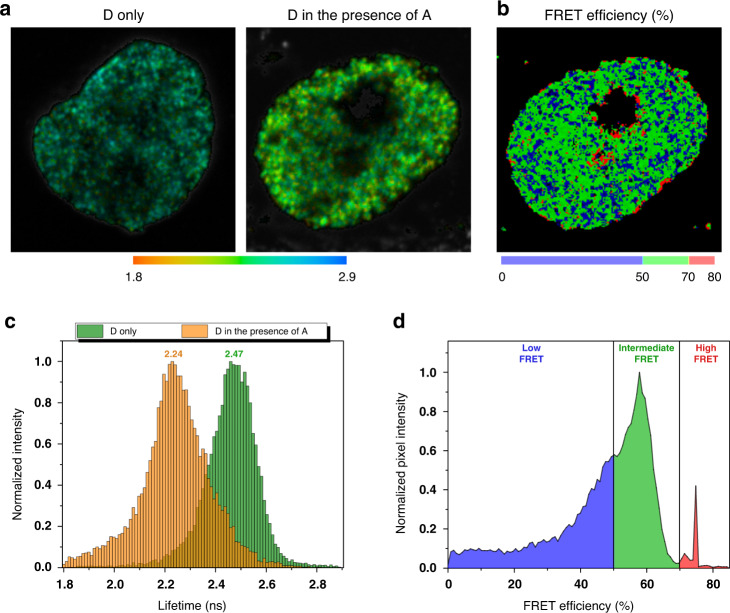
FLIM-FRET mapping of genomic DNA compaction in HeLa cell nucleus. The replicating cells were labeled in early and mid S-phase with AlexaFluor 546 (donor) and AlexaFluor 647 (acceptor) fluorophores correspondingly. **a** Representative images of AlexaFluor 546 lifetime distribution in the nucleus in the absence and in the presence of AlexaFluor 647. In the presence of acceptor, the fluorescence lifetime of the donor is shortened due to FRET (**b**). The FRET efficiency distribution in the cell nucleus calculated according to donor fluorophore lifetime distribution. Colormap was empirically defined from the FRET distribution histogram (shown on (**d**)). **c** Representative histograms of the donor lifetimes in the absence and in the presence of the acceptor. **d** FRET distributions histogram showing three populations (low, intermediate and high FRET), designated by corresponding colors. See Fig. S6 for additional examples

To assess the efficiency of the FLIM-FRET experimental approach, we labeled replicating DNA in early S-phase by simultaneous incubation with both CldU and IdU (Fig. 4a). Then, the sites of CldU and IdU incorporation were selectively labeled with AlexaFluor 546 and Alexa Fluor 647 fluorophores, which comprise an efficient FRET pair, as described in “Methods”.

In the cells which were simultaneously incubated with CldU and IdU the FLIM data show that in the presence of the acceptor, the average lifetime value of the donor was reduced from ~2.47 to ~2.24 ns, confirming the efficiency of FRET between the D and A fluorophores (Fig. 4c). Next, to characterize this proximity, we calculated the FRET efficiency in each pixel of the acquired image (Fig. 4b). The FRET maps were generated to localize low (below 50%), intermediate (50–70%) and high (over 70%) FRET efficiency regions using blue, green, and red discrete colors correspondingly (Fig. 4d). We found that high FRET zones (Fig. 4b; red color) were typically observed on the periphery of the nucleus and nucleolus, resembling the characteristic semi-circular arrangement of peri-nuclear and peri-nucleolar densely packed chromatin bodies known from classical light and electron microscopy studies^48^.

**Fig. 5:**
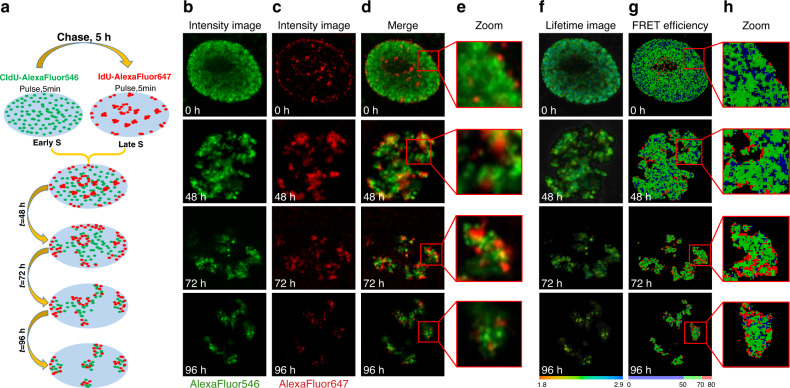
Simultaneous visualization of early and late S-phase replicating genomic DNA in the same cell nucleus by fluorescence imaging and the FLIM-FRET approach. **a** Schematics of approach: cultured cells sequentially labeled with CldU in early S-phase and with IdU in the late S-phase were chased into subsequent cell generations, enabling to visualize segregated chromosome territories with stained early and late S-phase labeled chromatin. **b**, **c** Cells were fixed at various intervals after the halogenated nucleotides pulses and stained for **b** CldU (AlexaFluor 546, green) and **c** IdU (AlexaFluor 647, red). Representative fluorescence intensity (**b**–**e**), lifetime images (**f**) and FRET efficiency (**g**, **h**) of the chromosome territories formation process after labeling of the early and late S replicating chromatin domains. Additional representative images are shown at Fig. S7

In further experiments, we validated a FLIM-FRET approach for probing the proximity between DNA fibers with different replication timing. In these experiments, we employed the cell-cycle synchronization protocol for selective labeling of early S-phase replicated chromatin, and chromatin replicated in the late S-phase^28^ with the FRET pair of fluorophores (AlexaFluor 546 and AlexaFluor 647). Fluorescence intensity and lifetime images were acquired either immediately after incorporation of halogenated nucleotides or several cell generations later, when individual chromosome territories could be clearly identified in the cell nucleus (Fig. 5).

Same as above, the DNA replication labeling pattern was consistent with the synchronization protocol, with early S-phase labeled sites scattered throughout the nucleoplasm, while the mid and late S-phase replicating DNA was concentrated in larger clusters, often located on the periphery of the nucleus and nucleolus (Fig. 5b–d). The top row on Fig. 5b and c shows representative distribution of the early S and late S-phase replicated chromatins domains. Same as in the experiments above, the segregation of labeled chromosome territories was already visible in cells, which were allowed to grow 48 h after the incubation with the halogenated nucleotides. In the cells grown for 72 and 96 h, the segregation was more pronounced enabling to identify individual chromosome territories, which facilitates image analysis. As expected, the characteristic distribution patterns of early and late S-phase replication sites were maintained through subsequent cell cycles^28^. Consistent with previous studies, we observed no significant overlap between early and late S-phase labeled chromatin (Fig. 5d, e)^28^.

At the same time, surprisingly, we documented a number of clear FRET clusters in the labeled chromatin (Fig. 5g, h), despite a lack of colocalization between the early and the late S-phase labeled DNA visible in the intensity images. Considering that a shortening of donor lifetime indicates a close proximity to acceptor, our data suggest that the clusters with a high FRET shown in red (Fig. 5g, h) contain both the early and the late S-phase replicating DNA segments co-compacted in the same chromatin fibers. At the same time, blue and green clusters on FLIM map represent chromatin regions wherein early S- and late-S labeled DNA sequences are spaced apart beyond the FRET distance threshold.

Overall, our pilot experiments provide a proof of concept for the unique applications of the FLIM-FRET approach in sensing of DNA compaction. While in our experiments we applied the immunolabeling protocol, the existing methodology enables replication labeling of chromatin in live cells by intracellular delivery of fluorescent nucleotides^30,31,50,53^. Hence, the FLIM approach is compatible with live cell experimental systems. Furthermore, labeling of both DNA strands may provide a unique mechanism for monitoring of DNA and RNA synthesis in real time (Fig. S8).

## Discussion

Herein we have proposed and validated two FLIM-based techniques for studying DNA compaction and gene activity. The first approach, which relies on the correlation between the fluorophore’s lifetime values and local RI, was applied to detect variations in nuclear chromatin compaction level throughout S-phase. The noticeable decrease in the mean lifetime up to ~230 ps was detected during the transition from early to mid and late S-phase. This lifetime shortening is indicative of a gradual increase in the local RI in the mid and late S-phase replicated chromatin as compared to that of the early S-phase replicated chromatin domains. Hence, we concluded that higher packing of DNA together with the recruitment of DNA-binding proteins led to a higher RI in mid to late S phase replicated CD, which in turn is manifested by a shorter fluorescence lifetime for the local fluorophores (Fig. 1c). An increase in the compaction density of mid and late S- replicated DNA could be expected from classic electron and light microscopy studies showing that at the beginning of S-phase replicates loosely compacted DNA, while densely packed clumps of heterochromatin replicate in the mid to late S-phase^29,30^. Nevertheless, FLIM approach described here enables quantitative measurements of variations in the density of early, mid, and late S- phase replicated chromatin.

While the FLIM approach described above is intended for fast and reproducible mapping of the chromatin packing density, the RI is not a nanoscopic value, which limits the spatial precision of the measurements. In comparison, the FLIM-FRET technique is inherently more sensitive with the capability for sensing of DNA compaction at the nanoscale. This technique has uncovered low, intermediate and high FRET zones, indicative of variations in chromatin compaction levels. Earlier, using fluorescence proteins fused to nucleosomes it was confirmed that the FRET efficiency correlates with the chromatin compaction density^39,54^. Hence, we interpret that the high FRET zones (Fig. 4; red color on FRET map) correspond to the highest degree of chromatin condensation, while intermediate (green color) and low FRET (blue color) values associate with less compacted chromatin structures. Furthermore, it is broadly accepted, that a dense compaction is a hallmark of transcriptionally silent chromatin, while active genes are typically present in the relaxed chromatin fibers. Thus, the FRET map provides generalized information on transcription activity of the fluorescence-labeled CDs.

An intriguing application of the FLIM-FRET approach is the probing of proximity and co-compaction between the DNA segments replicated in different windows of the S-phase. It has been long established that DNA replication occurs in relatively short, spatially separated stretches also known as replicons. Each chromatin domain accommodates up to 100 of such replicons, which are synchronously activated within a short interval of S-phase (45–60 min)^28,55^. In the studies using intensity-based fluorescence imaging, it was found that DNA segments replicated at different windows of the S-phase, are localized in separate CDs and do not mix together^28^. However, our understanding of correlation between DNA replication timing, proximity of replicons, and compaction is far from being complete. Up-to-date the arrangement and interactions of individual replicons within ~100 nm CDs is not known due to the limited resolution of conventional microscopy techniques. Furthermore, the extent of DNA separation between different CD is not clear. The intensity-based imaging techniques are designated for studies of large pools of DNA and could not interrogate the proximity of individual DNA fibers. In particular, gene compaction density is not constant, but fluctuates over time^30^. Moreover, the signal intensity strongly depends on the accessibility and binding of fluorophores, variable due to DNA compaction, that make a co-localization analysis even more complex. While it has been proposed that non-coding DNA sequences replicated in the late S-phase may loop out of their CD and participate in relocation and epigenetic regulation of active genes^56^ replicated in early S-phase, there is still a limited methodological platform to study this issue. We propose that fluorescence lifetime measurements offer a number of advantages over intensity-based techniques, as fluorophore lifetimes are independent on probe concentration, intensity fluctuations, and photobleaching^13,57^.

In order to demonstrate above mentioned advantages of FLIM-based technique, the simultaneous analysis of the same cells by conventional fluorescence imaging and FLIM-FRET method was performed. Primarily, the fluorescence intensity images support a view that the bulk of DNA replicated in the early and late S- phase is maintained in separate chromatin domains. However, in this study we detected FRET clusters in the labeled chromatin (Fig. 5g, h), suggesting a co-compaction of the early and the late S-phase replicating DNA segments. Interestingly, comparing the fluorescence intensity with the FLIM-FRET images for the same nuclear region, it is evident that FRET often occurs in weakly stained regions. This low signal intensity (Fig. 5e) makes it difficult to analyze a co-localization of the labeled chromosome material using conventional fluorescence imaging, however, FRET maps unambiguously identify the adjacent chromatin fibers (Fig. 5h).

Large variations in compaction of chromatin fibers significantly contribute to the regulation of gene expression, DNA replication and other genomic processes. Therefore, DNA compaction in the cell nucleus attracts broad interest in cell science, despite significant limitations of conventional methodology in characterization of chromatin fine structure. In conclusion, FLIM and FLIM-FRET techniques presented in this paper, provide new capabilities to this research field by enabling quantitative probing of chromatin compaction density and proximity of replication-labeled chromatin fibers in either live or fixed cells. Based on well-established methodology developed for staining of DNA during replication, sensing of RI by FLIM allows to map the variations in chromatin compaction throughout the cell nucleus. While the higher density of the late S-phase replicated chromatin has been long implied, the FLIM technique enables quantitative mapping of variations in the DNA compaction. Moreover, this FLIM assay indicates substantial differences in the packing of gene-rich pool of DNA replicated in the same window of early S-phase. In addition, the FLIM-FRET approach enables to study the mutual interactions of DNA segments with different replication timing labeled by donor-acceptor pair of fluorophores. Overall these optical approaches offer new directions in quantitative studies of genomic structure organization.

## Materials and methods

### Cell culture, synchronization, and Immunofluorescence staining

HeLa cells were cultured in advanced Dulbecco’s Modified Eagle Medium (DMEM, Life Technologies), supplemented with 3% fetal calf serum (FBS, Sigma), 1% glutamax (Life Technologies), and 1% antibiotic antimycotic solution (Sigma) at 37 °C in a humidified atmosphere containing 5% CO_2_. Cells were seeded on glass-bottom dishes (Mattek) and allowed to adhere for 24 h before synchronization. The cells were synchronized in the presence of 2 μg/ml aphidicolin, an inhibitor of DNA polymerase, for 12 h. After aphidicolin release and an additional 12 h growth, the cells were blocked at the G1/S border in the subsequent cell cycle by incubating with aphidicolin for 20 h. Following release from second aphidicolin block, the cells were incubated for either 1, 5, or 9 h until they entered the early mid or late S-phase, respectively. Then cells were pulse-labeled with halogenated nucleotides. In the single labeling experiments, synchronized cells were incubated with 10 μM -Bromo-2-Deoxy-Uridine (BrdU) for 5 min, fixed with 2% PFA/PBS solution for 12 min, extracted with 4 N HCl for 20 min and labeled with rat anti-BrdU antibody, followed with anti-rat AlexaFluor 546 secondary antibody. In the double labeling (pulse-chase-pulse) experiments, synchronized cells in the early S-phase were pulse-labeled for 5 min with 10 μM 5-chloro-2′-deoxyuridine (CldU), placed in a fresh medium for 5 or 9 h (depending on synchronization protocol), and pulsed again for 5 min with 10 μM 5-iodo-2′-deoxyuridine (IdU). The cells were then chased for 0, 48, 72, or 96 h before fixation. Antibody reactions were carried out as described^28,52^ using rat and mouse anti-BrdU antibodies, which selectively recognize CldU and IdU correspondingly. The fluorescence staining was performed using anti-rat AlexaFluor 546 (Invitrogen) and anti-mouse AlexaFluor 647 (Invitrogen) secondary antibodies. Corresponding lifetime images of genomic DNA distribution labeled with AlexaFluor 546 and AlexaFluor 647 acquired at key points of pulse-chase-pulse labeling procedure, are shown on Fig. S1. All images were acquired within 24 h after the cellular fixation.

### FLIM-FRET measuring and analysis

The fluorescence lifetime images were recorded using DCS-120 confocal laser scanning FLIM system based on a TCSPC module (SPC-150, Becker & Hickl, Berlin, Germany). The scanner was attached to an inverted microscope (ECLIPSE TE2000-E, Nikon, Japan). Single photon excited fluorescence from the sample was collected through a Plan APO 100×/NA1.4 oil immersion objective. The signals were detected by Hybrid Photon Detectors HPM-100-40 (Becker & Hickl, Berlin, Germany) that were connected to a TCSPC module (Becker & Hickl, Berlin, Germany). The excitation source was a picosecond super continuum (400–650 nm) laser with an acousto-optic tunable filter (AOTF) (SC400-4, Fianium, UK). The AlexaFluor 546 and Alexa Fluor 647 fluorescence were excited by 545 and 640 nm, respectively. In order to avoid significant pile-up error in all our measurements, the peak count rate never exceeded the recommended 10% of the excitation rate. The fluorescence signals were collected using band-pass filters ET590/50 for AlexaFluor 546, and ET700/75 for AlexaFluor546 and AlexaFluor647 (Chroma Technology Corp., Bellows Falls, USA). All images were acquired at 256 × 256 pixels. The signal acquisition time was around 60 s. The number of nuclei analyzed for each particular experiment was not less than 10. The analysis of the FLIM data, including lifetime and FRET efficiency calculation was performed using SPCImage (Becker & Hickl, Berlin, Germany) as described in the manufacturer’s protocols^13^.

As fluorescence decay of the AlexaFluor 546 can be fitted with a single exponential function with high precision (the reduced χ^2^ value close to 1), a mono-exponential model was used for lifetime calculation in the single -labeling experiments. During data analysis the threshold was set to 30 in all data sets (Figs. S9–10). The binning factor was set to 1. For the FRET experiments, DNA was labeled with AlexaFluor 546 and AlexaFluor 647, which serve as a donor (D) and acceptor (A) correspondingly. The D emission and A absorption spectra, together with the spectral overlap integral J(λ), are shown in Fig. S2. In order to check the background autofluorescence level in cells nuclei, control experiments with non-labeled samples were performed. In these experiments with non-labeled samples, no autofluorescence above the threshold level was detected.

The presence of the acceptor enables energy transfer from the donor to the acceptor, which, consequently leads to a decrease of the donor lifetime. In control experiments, the averaged fluorescence lifetime values of the donor without the acceptor and in the presence of the acceptor were calculated using the single-exponential lifetime analysis (Fig. S9c), in order to demonstrate that the averaged lifetime of the donor shortens in presence of the acceptor due to FRET (Fig. 4a and c). We did not observe any correlation of measured lifetime with intensity, other than higher measurements deviation (Fig. S9a, b). However, in a more precise model, fluorescence decay curves for the donor-acceptor pair are presented as a linear sum of the two components:

$f\left( t \right) = a_1e^{ - (t/\tau _{\rm{fret}})} + a_2e^{ - (t/\tau _0)}$, where the slow component (τ_0_) corresponds to the non-interacting donor and the faster component (τ_fret_) corresponds to the donor interacting with the acceptor^13,58^. Therefore, for the FRET experiments, fluorescence decay of the donor was analyzed using the double-exponential lifetime model (Fig. S9c) and the FRET efficiency was correspondingly calculated according to equation: ${{{\mathrm{E}}}}_{\rm{fret}} = 1 - (\tau _{\rm{fret}}/\tau _0)$^58^.

## Supplementary information

Supplementary Information
