# Supplementary material for: Fluorescence lifetime imaging for studying DNA compaction and gene activities
Source: Light Sci Appl. 2021 Nov 2;10:224. doi: 10.1038/s41377-021-00664-w (PMC8563720; doi:10.1038/s41377-021-00664-w)
Supplement: Supplementary file 1 — Supplementary Information [file 41377_2021_664_MOESM1_ESM.docx]

**Supplementary Information for**

**Fluorescence lifetime imaging for studying DNA compaction and gene activities**

Svitlana M. Levchenko^1,2^, Artem Pliss^3^, Xiao Peng^1^, Paras N. Prasad^3*^, Junle Qu^1*^

^1^ Key Laboratory of Optoelectronic Devices and Systems of Ministry of Education and Guangdong Province, College of Physics and Optoelectronic Engineering, Shenzhen University, Shenzhen, Guangdong 518060, China

^2^ Department of Cell Biophysics, Faculty of Biochemistry, Biophysics and Biotechnology, Jagiellonian University, 30-387 Krakow, Poland

^3^ Institute for Lasers, Photonics and Biophotonics, University at Buffalo, State University of New York, Buffalo, NY 14260-3000

Correspondence:

*Paras N. Prasad: pnprasad@buffalo.edu

*Junle Qu: jlqu@szu.edu.cn

**Supplementary Figures**


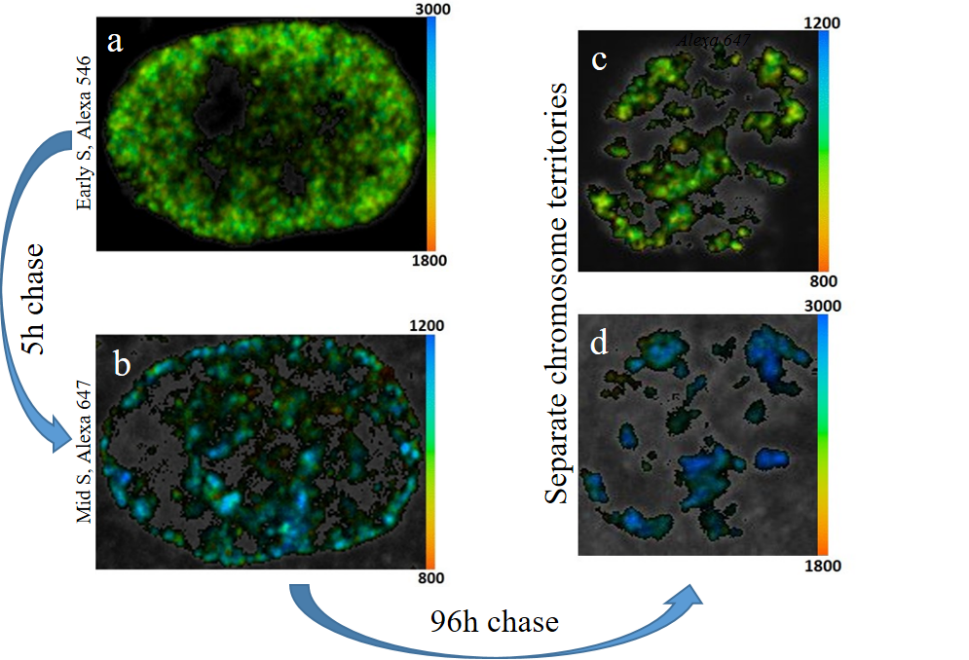


**Figure S1.** Lifetime images of genomic DNA distribution labeled with AlexaFluor546 (donor) and AlexaFluor647 (acceptor) acquired at key points of pulse-chase-pulse labeling procedure. In the double labeling experiments, synchronized cells in the early S-phase were pulse-labeled for 5 min with 5-chloro-2′-deoxyuridine (a), placed in a fresh medium for 5 h, and pulsed again for 5 min with 5-iodo-2′-deoxyuridine (b). The cells were then chased 96 h to allow segregation of chromosome territories into separate labeled regions (c, d). The fluorescence staining was performed using anti-rat AlexaFluor546 and anti-mouse AlexaFluor647 secondary antibodies


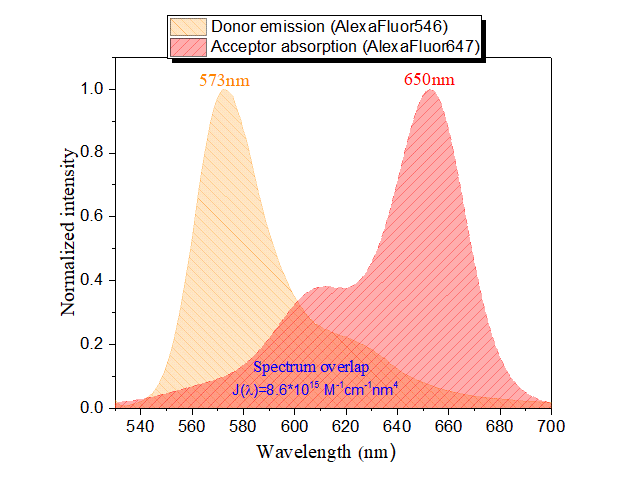


**Figure S2.** Donor emission (yellow) and acceptor absorption (red) spectra together with calculated spectral overlap integral J(λ). The spectral overlap integral J(λ)=$\int_{0}^{\infty} F_{D}(\lambda)\varepsilon_{A}(\lambda)\lambda^{4}d\lambda$ (where F_D_- normalized emission spectrum of the donor, ε_A_- absorption coefficient of the acceptor, λ-wavelength), obtained from published spectra on the manufacturer website: <https://www.thermofisher.com/order/fluorescence-spectraviewer#!/>


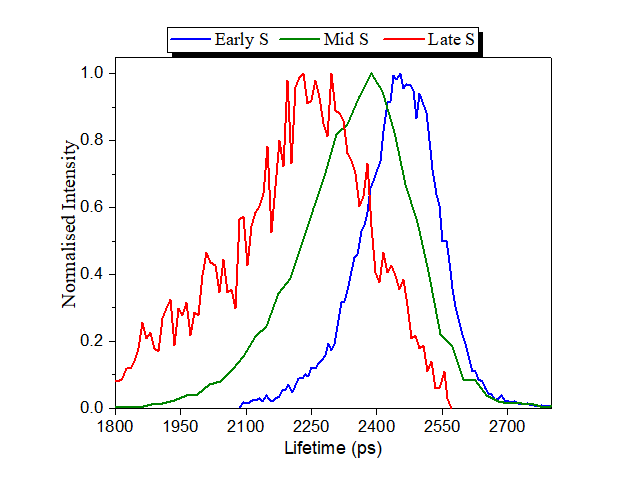


**Figure S3.** Representative fluorescence lifetime histograms of AlexaFluo546 labeled genomic DNA in early, mid, and late S-phase


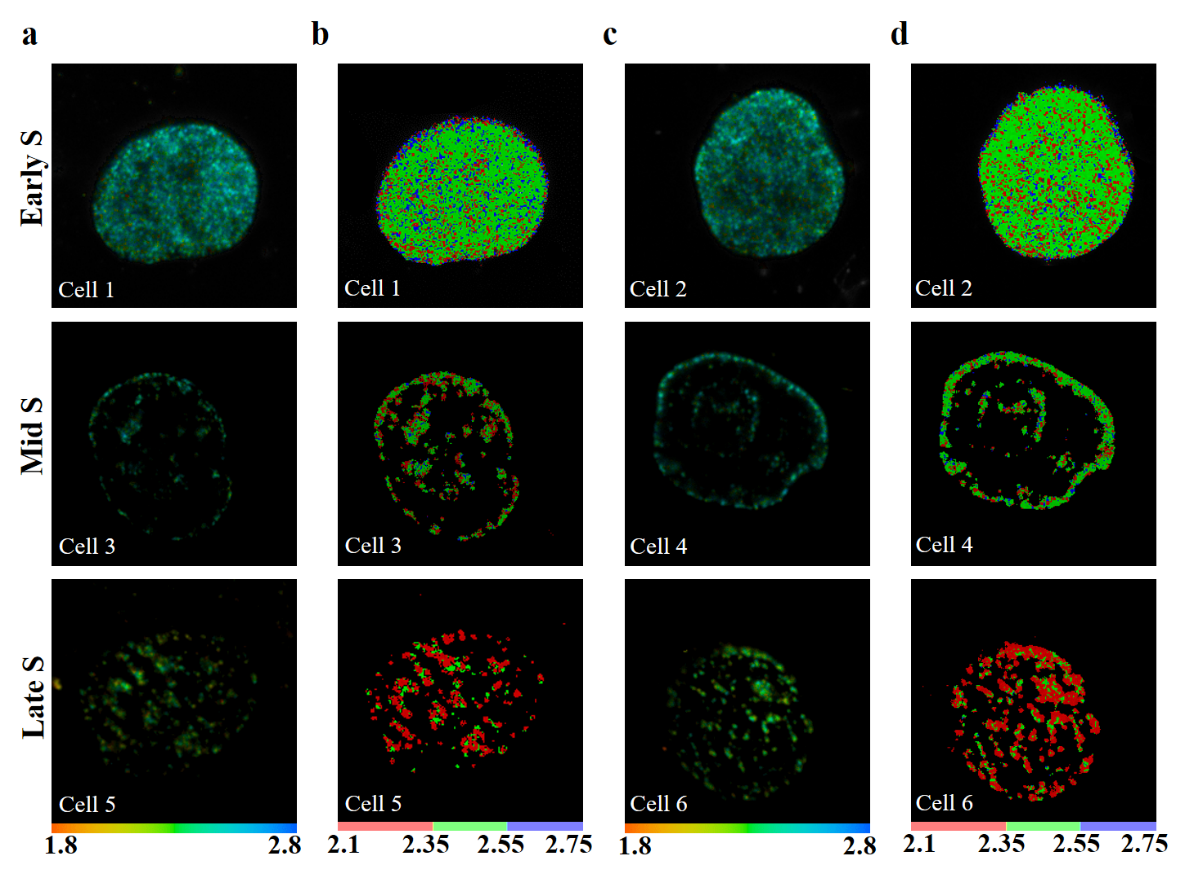


**Figure S4**. (a, c) Representative fluorescence lifetime images of DNA replication sites labeled with AlexaFluor 546 at the sequential stages of S-phase: early S – top row (cell 1 and 2), mid S – middle row (cell 3 and 4) and late S – bottom row (cell 5 and 6). (b, d) Color-coded mapping of the fluorescence lifetimes throughout cell nucleus


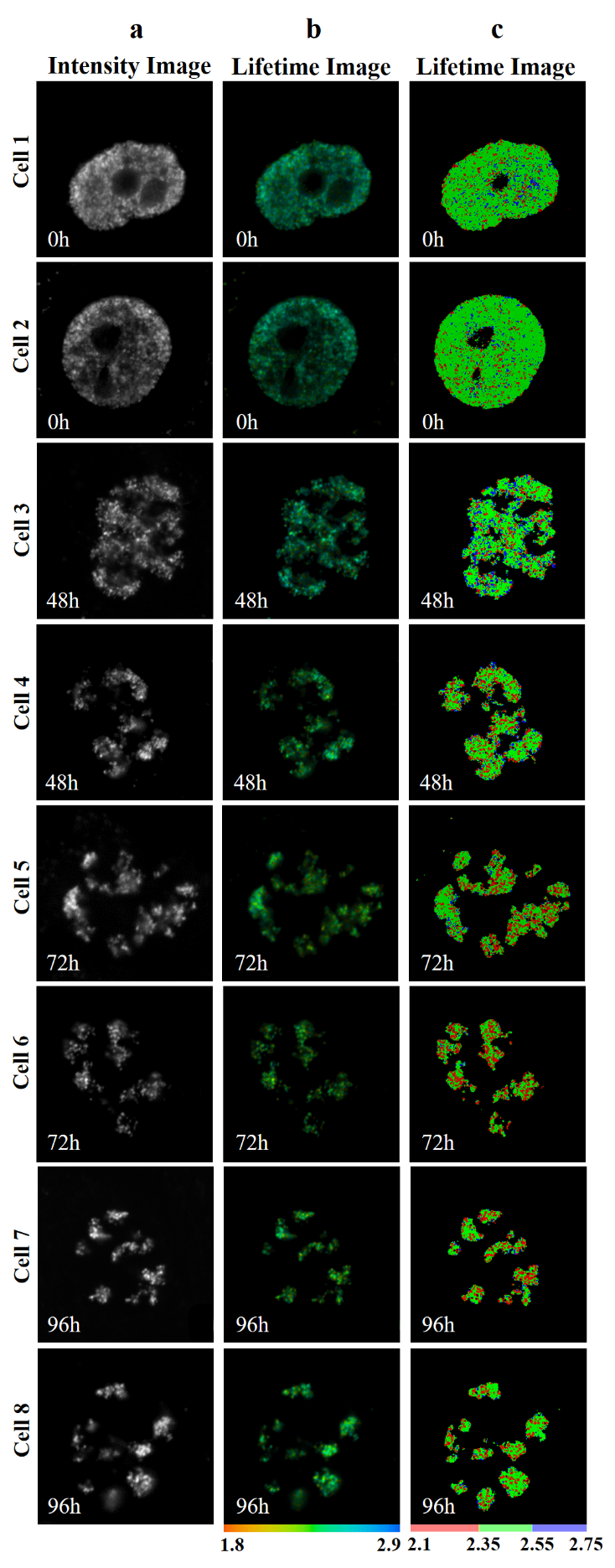


**Figure S5.** Fluorescence intensity (a), continuous (b) and discrete (c) fluorescence lifetime images of the early-S-phase replicated chromatin. Cells were labeled with a 5 min pulse of BrdU and chased in subsequent cell generations. The time passed after the BrdU pulse is indicated. Images from top to bottom show progressive segregation of the labeled chromosome territories


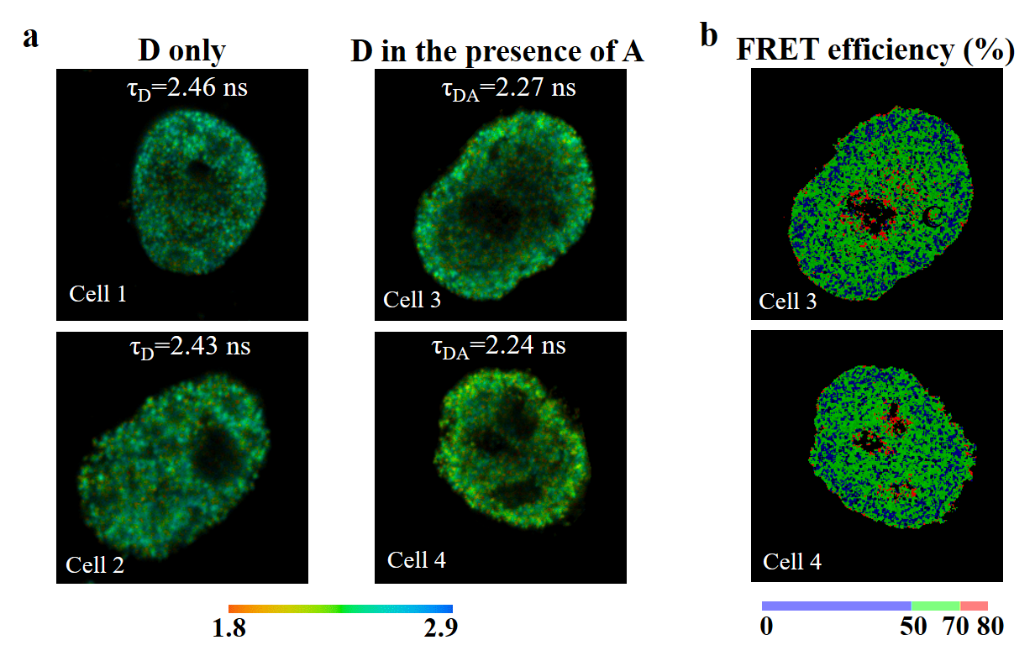


**Figure S6.** (a) AlexaFluor 546 (Donor) lifetime distribution in the nucleus in the absence (Cell 1 and 2) and in the presence (Cell 3 and 4) of AlexaFluor 647 (Acceptor). (b) The FRET efficiency was calculated according to Donor fluorophore lifetime distribution (Cell 3 and 4)


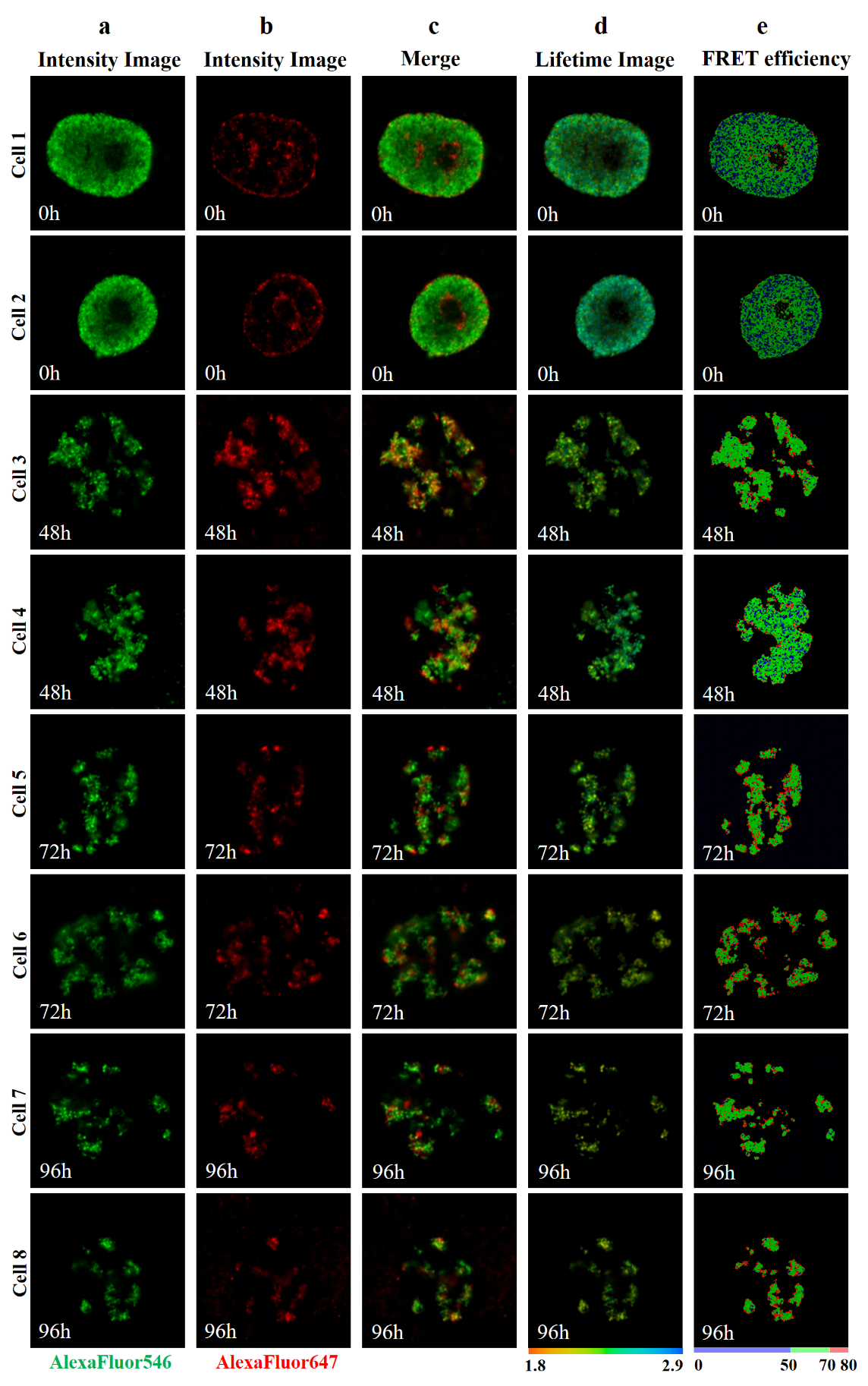


**Figure S7.** Simultaneous visualization of the early- and late S-phase replicating genomic DNA in the same cell nucleus by fluorescence imaging and the FLIM-FRET approach. Fluorescence intensity images (a-c), lifetime images (d) and FRET efficiency (e) are shown. Data characterize segregation of the chromosome territories after sequential labeling of the early and late S replicating chromatin domains with AlexaFluor546 and AlexaFluor647, correspondingly


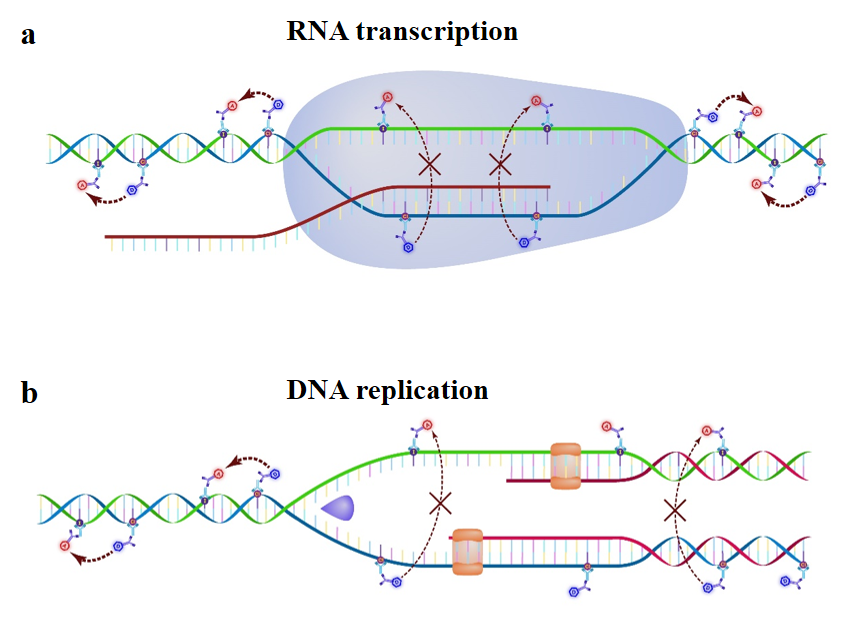


**Figure S8.** Schematic illustration of potential application of FLIM-FRET for monitoring of DNA and RNA synthesis in real time.

A combination of super-resolution microscopy and FLIM/FRET technique may enable probing of nucleic acid synthesis in real-time. While the protocols of this approach are currently under development, we outline here this new concept of optical biosensing.

We propose that the donor and the acceptor fluorophores are incorporated into the complementary DNA strands, during the S-phase in consequent cell cycles. Under these conditions FRET occurs between the donor and the acceptor fluorophores, when the DNA strands are arranged in the double helix structure. However, during certain genomic events, such as RNA synthesis (a) or DNA replication (b) the double helix unwinds leading to disruption of FRET. We thus propose that FLIM/FRET provides a platform for real time monitoring of DNA and RNA synthesis, at a level of single DNA replication site or transcription factory


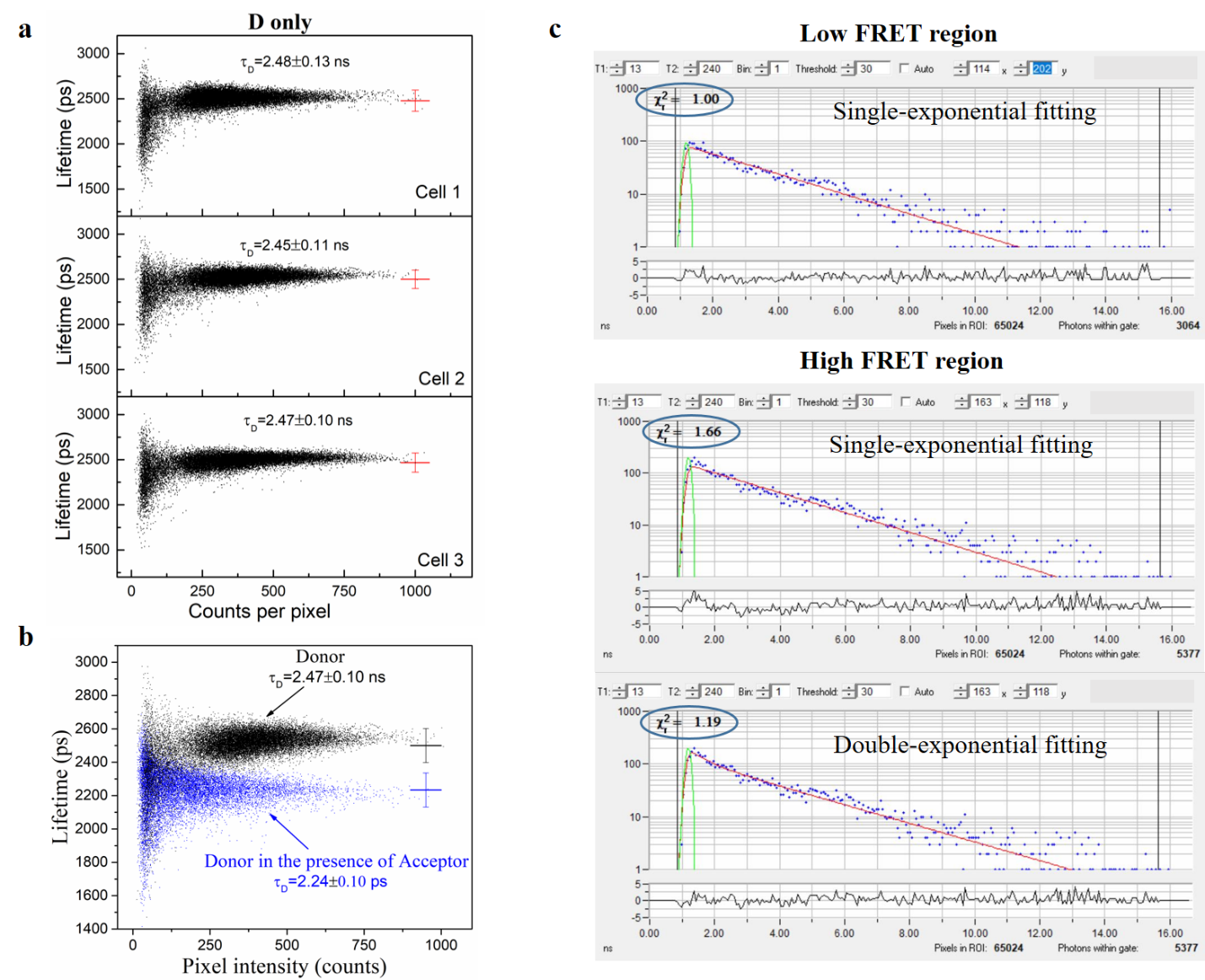


**Figure S9.** Scattered plots illustrate dependence between per-pixel fluorescence lifetimes of Donor fluorophore (AlexaFluor 546) and corresponding fluorescence intensities. The pixel intensity threshold was set to 30 counts to exclude contributions of autofluorescence and fluorescence background from unwashed probe (a). Representative lifetimes vs intensities pixel scatter plots generated for cells stained with either Donor fluorophore alone (black) or combination of Donor and Acceptor (AlexaFluor 647) fluorophores (blue) (b). Error bars denote mean ± SD. Illustration of the donor’s fluorescence decays fitting for the pixels in the low and high FRET efficiency regions (c). In case when cells labeled with Donor fluorophore alone or in a low FRET region the fluorescence decay of the AlexaFluor 546 is fitted with a single exponential function with high precision (χ^2^ ≈ 1), while fluorescence decay curve of the Donor fluorophore in high FRET region requires double-exponential lifetime model as indicated by corresponding χ^2^ values


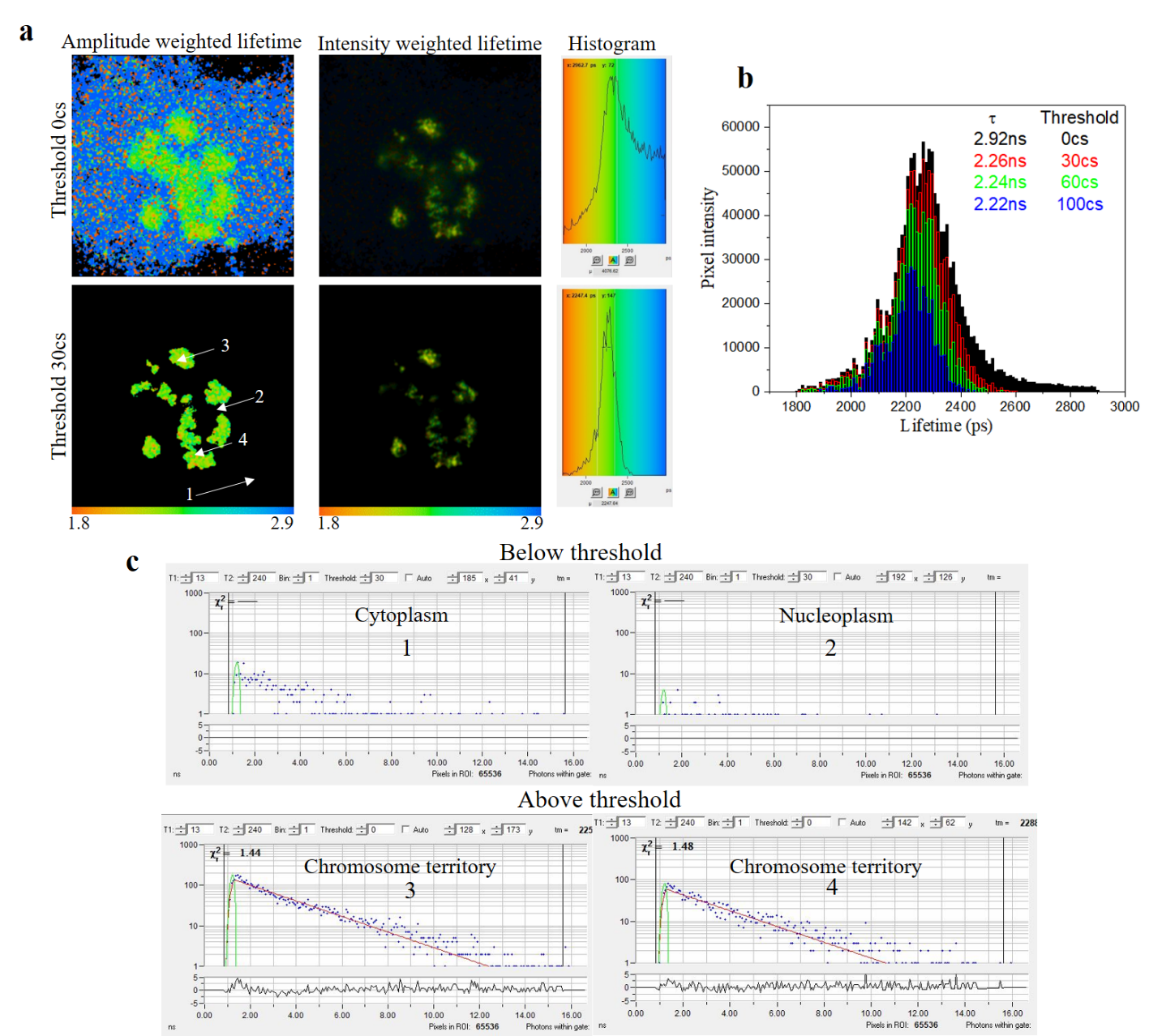


**Figure S10**. Selection of optimal background threshold for FLIM-FRET datasets. Representative amplitude and intensity weighted lifetime images together with lifetime histograms for the same cell, which labeled with Donor and Acceptor fluorophores, before and after thresholding (a). Donor’s lifetime histogram obtained with different threshold values (b). Removal 20-30 counts of background noise results in shortening of averaged fluorescence lifetime values. Further increase of threshold from 30 to 60 and 100 cs does not significantly affect mean lifetime. Representative fluorescence decay curves in selected spots in cytoplasm, nucleoplasm and chromosome territories. Note that nonlabelled regions in cytoplasm (1) and nucleus (2) were below 30 counts threshold and therefore not analyzed (c)
